# Basal Forebrain Volume Predicts Disease Conversion in Prodromal Synucleinopathy

**DOI:** 10.1002/mdc3.70242

**Published:** 2025-07-22

**Authors:** Lachlan Churchill, Ajay Konuri, Anna Ignatavicius, Jack Anderson, Simon J.G. Lewis, Elie Matar

**Affiliations:** ^1^ Faculty of Medicine and Health, Central Clinical School University of Sydney Sydney New South Wales Australia; ^2^ Faculty of Medicine, Health and Human Sciences, Macquarie Medical School and Macquarie University Centre for Parkinson's Disease Research Macquarie University Sydney New South Wales Australia; ^3^ Centre for Integrated Research and Understanding of Sleep (CIRUS) Woolcock Institute for Medical Research Sydney New South Wales Australia; ^4^ Department of Neurology Royal Prince Alfred Hospital Camperdown New South Wales Australia; ^5^ Brain and Mind Centre University of Sydney Camperdown Australia

**Keywords:** rapid eye movement sleep (REM) sleep, prodromal, Parkinson's disease, structural magnetic resonance imaging

## Abstract

**Background:**

Isolated rapid eye movement sleep behavior disorder (iRBD) is a prodromal stage of Parkinson's disease (PD) and dementia with Lewy bodies (DLB). The basal forebrain (BF), a key cholinergic structure, is a site of known pathology in later stages of Lewy body disorders. Although bilateral BF atrophy has been linked to cognitive decline in iRBD, its potential role in predicting phenoconversion to PD and DLB remains unclear.

**Objectives:**

The aims were to examine BF gray matter volume differences between iRBD patients and healthy controls, and evaluate their utility as predictors of phenoconversion to PD or DLB. Exploratory post hoc analyses were also conducted to explore the lateral‐specific effects of BF atrophy in relation to disease conversion.

**Methods:**

We assessed 41 participants with polysomnography‐confirmed iRBD and 38 healthy controls using baseline T1‐weighted magnetic resonance imaging (MRI) and longitudinal clinical assessments. Gray matter volumes of the left and right BF were compared between groups. Cox proportional hazards models examined baseline BF volumes as predictors of phenoconversion risk to PD and DLB.

**Results:**

Although no significant group differences in BF volume were found, lower BF volume was associated with poorer global cognition in iRBD. Bilateral BF atrophy predicted increased risk of phenoconversion to either PD or DLB. An exploratory post hoc analysis revealed that left BF atrophy specifically predicted conversion to DLB, whereas right BF volume did not.

**Conclusion:**

Bilateral BF atrophy may represent an early biomarker of phenoconversion in iRBD, with left‐sided atrophy potentially indicating increased risk for DLB. These findings highlight the prognostic value of BF degeneration in prodromal synucleinopathies.

Rapid eye movement (REM) sleep behavior disorder (RBD) is a parasomnia marked by the loss of skeletal muscle atonia during REM sleep resulting in dream enactment behaviors.^1^ Isolated RBD (iRBD) is now recognized to be an early manifestation of α‐synucleinopathies,^2^ with approximately 75% of individuals converting to Parkinson's disease (PD) or dementia with Lewy bodies (DLB) over a 12‐year interval.^3^ As such, longitudinal tracking of iRBD provides a unique opportunity to investigate early neurodegenerative signatures preceding clinical diagnosis that may potentially predict phenoconversion and future symptom development.^4^

Mild cognitive impairment (MCI) is frequently reported in iRBD, affecting nearly 50% of individuals.^5^ Cognitive deficits are particularly pronounced in attentional, executive function and visuospatial domains, which are hallmark features of PD and DLB.^6^ Particularly, the presence of MCI in iRBD has been associated with an increased risk of future dementia development and serves as a strong predictor of conversion to α‐synucleinopathies.^7^, ^8^ Furthermore, in established PD, the presence of RBD is associated with a greater burden of non‐motor symptoms and more rapid cognitive decline compared to those without RBD.^9^, ^10^

Recent studies have highlighted the cholinergic basal forebrain (BF) as a key structure affected in the pathological progression of both PD and DLB.^11^, ^12^, ^13^ The BF is integral to cognitive processing, providing widespread cholinergic projections to the majority of the neocortex and limbic system.^12^ Reductions in this cortical cholinergic innervation have been demonstrated in iRBD and more severely in DLB and PD dementia,^13^, ^14^, ^15^ contributing to the development of core non‐motor symptoms such as visual hallucinations and cognitive decline.^10^, ^16^ Similarly, structural magnetic resonance imaging (MRI) studies have consistently demonstrated that atrophy in the cholinergic BF is strongly associated with reduced cross‐sectional and longitudinal cognitive performance in iRBD^17^, ^18^, ^19^ and an increased risk of developing dementia in PD.^20^, ^21^, ^22^, ^23^

The lateralization of dopaminergic degeneration is well documented in Lewy body disorders,^24^ serving as a focal point in theories of PD pathogenesis^25^, ^26^ and accounting for the onset and asymmetrical presentation of motor symptoms.^27^ Interestingly, emerging evidence now suggests that cholinergic degeneration may also occur asymmetrically in both PD and DLB,^14^, ^28^ although the precise functional and clinical implications of this lateralization remain relatively unknown. To date, a contemporaneous study has demonstrated a predictive association between BF volume and DLB phenoconversion in iRBD.^29^ However, the role of asymmetry in this cholinergic BF degeneration, and its potential relationship to disease conversion and disruption of specific cognitive domains, remains largely unexplored.

To investigate this, we first compared BF volume between iRBD patients and healthy controls (HC) and then assessed its relationship with clinical and cognitive measures in iRBD. Leveraging longitudinal clinical data, we next applied Cox proportional hazards models to determine the prognostic significance of BF volume in predicting the transition to any synucleinopathy (PD or DLB), in addition to DLB specifically. This latter analysis was motivated by the established association between cholinergic deficits and cognitive impairment, implying the possibility of BF volume emerging as a relevant marker for dementia‐specific risk in iRBD. Additionally, we conducted an exploratory post hoc survival analysis to examine whether lateralized BF volumes differentially predicted conversion to DLB and PD separately.

## Patients and Methods

### Participants

Forty‐one participants with iRBD and 38 age‐matched HC were recruited from the Parkinson's Disease Research Clinic at the University of Sydney. REM sleep without atonia was identified by sleep specialists using video polysomnography, adhering to the International Classification of Sleep Disorders‐III criteria.^1^ A clinical diagnosis of iRBD was subsequently confirmed by neurologists following the guidelines outlined by the American Association of Sleep Medicine III.^30^ The study was approved by the local ethics committee in accordance with the Declaration of Helsinki.

### Clinical Assessments

Motor function was assessed using the Movement Disorders Society Unified Parkinson's Disease Rating Scale, Part III (MDS‐UPDRS III).^31^ A comprehensive battery of domain‐specific cognitive assessments and standardized questionnaires were administered to evaluate both cognitive performance and non‐motor symptom severity. Global cognition was assessed using the Montreal Cognitive Assessment (MoCA), whereas visuospatial function was measured using the Clock Drawing Test. Executive functioning was evaluated using the Trail Making Test Part B (TMT‐B) and the Color‐Word Interference Test (subtests 3 and 4) from the Delis–Kaplan Executive Function System. Attention and working memory were assessed using the Digit Span Backward subtest of the Wechsler Adult Intelligence Scale. Verbal learning and memory were examined using the Rey Auditory Verbal Learning Test and the Logical Memory subtest from the Wechsler Memory Scale‐Third Edition. Language function was assessed using the Controlled Oral Word Association Test.

Non‐motor symptoms were evaluated using the following questionnaires: sleep disturbances were evaluated using the Scales for Outcomes in Parkinson's Disease‐Sleep (SCOPA‐Sleep), which includes both nighttime (SCOPA‐Sleep Night) and daytime (SCOPA‐Sleep Day) components. Daytime sleepiness was also further assessed using the Epworth Sleepiness Scale. Symptoms of RBD were measured using the REM Sleep Behavior Disorder Screening Questionnaire. Mood symptoms were assessed using the Hospital Anxiety and Depression Scale (HADS), which includes separate subscales for anxiety (HADS‐A) and depression (HADS‐D). Olfactory function was evaluated using the Sniffin' Sticks 16‐item odor identification test.

### MRI Acquisition and Processing

Participants underwent a structural T1‐weighted MRI scan within 1 year of their clinical assessments. Imaging data were collected on a 3‐T MRI scanner (General Electric, Boston, MA, USA). Sagittal three‐dimensional T1‐weighted structural images were obtained with an echo time (TE) of 2.7 ms, a repetition time (TR) of 7.2 ms, an acquisition matrix of 256 × 256, 200 slices, and a slice thickness of 1 mm.

T1‐weighted images were segmented using the recon‐all pipeline of the cross‐sectional stream of FreeSurfer, version 7.1.1.^32^ The bilateral BF was segmented using ScLimbic (mm^3^), a deep‐learning toolbox designed for FreeSurfer (Fig. S1).^33^ BF volumes were adjusted for estimated total intracranial volume using the residual correction method (Data S1).^34^

### Statistical Analysis

Statistical analyses were conducted using MATLAB (Release 2023b, MathWorks, Inc., Natick, MA, USA). Differences in BF volumes between HC and iRBD were assessed using a general linear model with age, sex, and education as covariates. Correlations between BF volumes and clinical or cognitive testing were assessed within the iRBD cohort using Spearman's rank correlation analysis. No covariates were included in the correlation analysis. Multiple comparisons in cognitive domain–specific tests were corrected using the Benjamini–Hochberg procedure to control for the false discovery rate with *P* < 0.05.

Participants were longitudinally tracked from baseline imaging to last‐recorded clinical visit, with date of phenoconversion documented and stability of diagnosis confirmed (10 PD and 7 DLB converters total). Two survival models were constructed: one examining conversion to any α‐synucleinopathy (PD or DLB vs. iRBD nonconverters) and another specifically for dementia‐specific conversion (DLB vs. nondemented PD and iRBD nonconverters). BF volume was entered as a continuous predictor in all survival models. Follow‐up time was measured in months (mean = 41, maximum = 126). Age was included as the only covariate to maintain a 5–10 events per variable ratio. Hazard ratios (HR) with 95% confidence intervals (CI) were calculated, and model fit was assessed using log‐likelihood ratio tests, with significance set at *P* < 0.05.^35^ For visualization purposes only, Kaplan–Meier curves were constructed by splitting groups into high and low BF volumes around the median value.

## Results

### Clinical Data

A comparison between the iRBD and HC groups revealed no significant differences in age or education (Table 1). However, the iRBD cohort included a significantly greater proportion of men (*P* < 0.001). Patients with iRBD performed worse on the MoCA, TMT‐B and Clock Drawing Test compared to the control group (*P* = 0.012 and *P* = 0.037, respectively). Furthermore, olfactory and motor function (Sniffin' Sticks and MDS‐UPDRS III) were significantly more impaired in iRBD patients (*P* < 0.001).

**TABLE 1:** Population demographics

Variable	Control (n = 38)	iRBD (n = 41)	*P*‐value
Clinical demographics			
Age (yr)	66.9 (7.6)	65.9 (6.7)	0.53
Education (yr)	14.2 (2.8)	13.2 (3.0)	0.095
Disease duration (yr)		1.9 (2.2)	
Disease conversion		10 PD, 7 DLB	
Sex	17 male, 21 female	34 male, 7 female	**<0.001**
Questionnaires			
SCOPA‐Sleep Night	4.1 (3.6)	2.7 (3.3)	0.17
SCOPA‐Sleep Day	2.3 (2.1)	2.7 (3.6)	0.63
Epworth Sleepiness scale	5.2 (3.2)	7.1 (5.5)	0.12
RBD screening questionnaire	2.6 (2.0)	7.5 (3.2)	**<0.001**
HADS anxiety	2.6 (2.2)	3.1 (3.4)	0.50
HADS depression	1.8 (1.8)	2.6 (3.4)	0.24
Cognitive measures			
Montreal Cognitive Assessment	28.0 (2.0)	26.7 (2.6)	**0.012**
Executive function (TMT‐B)	0.6 (0.6)	0.1 (1.1)	**0.037**
Visuospatial function (Clock Drawing Test)	10 (0)	9.7 (0.5)	**0.046**
Attention and working memory (Digit Span Backward)	12.5 (2.8)	11.8 (2.9)	0.32
Learning and memory (RAVLT)	−0.92 (1.7)	−1.1 (1.9)	0.77
Learning and memory (WMS‐III)	11.3 (0.9)	10.9 (1.1)	0.60
Language (COWAT)	0.50 (0.9)	0.18 (1.1)	0.24
Inhibition (D‐KEFS CWIT 3)	11.7 (3.1)	11.1 (2.6)	0.48
Switching and cognitive flexibility (D‐KEFS CWIT 4)	12.2 (2.5)	11.3 (2.8)	0.23
Sensory/motor			
Olfactory function (Sniffin' Sticks)	10.1 (1.7)	7.1 (3.0)	**<0.001**
MDS‐UPDRS III	1.3 (1.8)	9.2 (8.3)	**<0.001**
Corrected gray matter volume			
Left BF (mm^3^)	305.9 (42.7)	305.3 (41.2)	0.30
Right (mm^3^)	322.1 (47.7)	321.4 (37.8)	0.26
Bilateral BF (mm^3^)	627.37 (81.3)	627.32 (74.2)	0.21

*Notes*: Values are shown as mean (standard deviation). Participants were matched for age and education, but the ratio of males to females differed between groups. *P*‐values were calculated using nonparametric permutation testing for clinical, questionnaire, cognitive, and sensory/motor measures, whereas χ^2^ test was implemented for sex differences. *P‐*values for the left and right BF volumes were calculated using a general linear model with age, sex, and education as covariates. Values in bold indicate statistically significant group differences between iRBD and healthy controls (*P < 0.05, two‐sided)* Montreal Cognitive Assessment and Clock Drawing Test are reported as raw scores. Digit Span Backward, WMS‐III, D‐KEFS CWIT subtests 3 and 4, and RAVLT are reported as standardized scores normed for age and education. COWAT and TMT‐B are reported as *z*‐scores adjusted for age and education.

Abbreviations: iRBD, isolated rapid eye movement sleep behavior disorder; PD, Parkinson's disease; DLB, dementia with Lewy bodies; SCOPA, Scales for Outcomes in Parkinson's Disease; HADS, Hospital Anxiety and Depression Scale; TMT‐B, Trail Making Test Part B; RAVLT, Rey Auditory Verbal Learning Test; WMS‐III, Wechsler Memory Scale‐Third Edition; COWAT, Controlled Oral Word Association Test; D‐KEFS, Delis–Kaplan Executive Function System; CWIT, Color‐Word Inference Test; MDS‐UPDRS III, Movement Disorders Society‐Unified Parkinson's Disease Rating Scale, Part III; BF, basal forebrain.

### Group Comparisons and Correlations in BF Volume in iRBD

There were no significant differences in BF volumes in iRBD compared to HCs (Table 1). Spearman's correlations demonstrated that BF volumes were significantly negatively correlated with age (B: rho = −0.465, *P =* 0.00218; L: rho = −0.44, *P* = 0.003; and R: rho = −0.43, *P* = 0.005) (Table 2). Reduced BF volumes were also significantly correlated with lower MoCA scores (B: rho = 0.411, *P* = 0.010; L: rho = 0.37, *P* = 0.022; and R: rho = 0.42, *P* = 0.009). There was a trending correlation between the TMT‐B and BF volumes; however, this did not reach significance (B: rho = 0.362, *P* uncorrected = 0.028; L: rho = 0.33, *P* uncorrected = 0.048; and R: rho = 0.34, *P* uncorrected = 0.052).

**TABLE 2:** Spearman's rank correlation analysis of clinical scores with BF volumes

Cognitive tests	Left BF volume	Right BF volume	Bilateral BF volume
Montreal Cognitive Assessment	**rho = 0.370, *P* = 0.022**	**rho = 0.421, *P* = 0.009**	**rho = 0.411, *P* = 0.010**
Domain‐specific cognition			
Executive function (TMT‐B)	rho = 0.323, *P* = 0.052, p_FDR_ = 0.412	rho = 0.328, *P* = 0.0481, p_FDR_ = 0.336	rho = 0.362, *P* = 0.0279, p_FDR_ = 0.210
Attention and working memory (Digit Span Backward)	rho = 0.0100, *P* = 0.954, p_FDR_ = 0.954	rho = 0.0520, *P* = 0.766, p_FDR_ = 0.766	rho = 0.0200, *P* = 0.909, p_FDR_ = 0.908
Inhibition (D‐KEFS CWIT 3)	rho = 0.118, *P* = 0.501, p_FDR_ = 0.582	rho = 0.149, *P* = 0.390, p_FDR_ = 0.593	rho = 0.168, *P* = 0.334, p_FDR_ = 0.534
Inhibition and switching (D‐KEFS CWIT 4)	rho = 0.161, *P* = 0.356, p_FDR_ = 0.582	rho = 0.207, *P* = 0.234, p_FDR_ = 0.545	rho = 0.218, *P* = 0.207, p_FDR_ = 0.414
Learning and memory (RAVLT)	rho = 0.333, *P* = 0.225, p_FDR_ = 0.582	rho = 0.348, *P* = 0.203, p_FDR_ = 0.545	rho = 0.450, *P* = 0.0917, p_FDR_ = 0.244
Learning and memory (WMS‐III)	rho = −0.149, *P* = 0.391, p_FDR_ = 0.582	rho = 0.136, *P* = 0.437, p_FDR_ = 0.593	rho = −0.0621, *P* = 0.723, p_FDR_ = 0.826
Language (COWAT)	rho = 0.113, *P* = 0.510, p_FDR_ = 0.582	rho = 0.114, *P* = 0.509, p_FDR_ = 0.593	rho = 0.0972, *P* = 0.572, p_FDR_ = 0.763
Clinical measures			
MDS‐UPDRS III	rho = −0.229, *P* = 0.186	**rho = −0.453, *P* = 0.006**	rho = −0.330, *P* = 0.053
Olfactory function (Sniffin' Sticks)	rho = 0.102, *P* = 0.353	rho = 0.223, *P* = 0.253	rho = 0.232, *P* = 0.234
Demographic measures			
Age	**rho = −0.446, *P* = 0.003**	**rho = −0.433, *P* = 0.005**	**rho = −0.465, *P* = 0.002**
Sex	rho = −0.301, *P* = 0.056	rho = −0.225, *P* = 0.157	rho = −0.307, *P* = 0.051

*Notes*: Spearman's rank correlation analysis of clinical scores with BF volumes. The table presents Spearman's correlation coefficients (rho) and *P*‐values for the relationships between basal forebrain volumes (left, right, and bilateral) and various clinical, demographic, and cognitive measures. Bolded values indicate statistically significant correlations (*P < 0.05, two sided)*. For domain‐specific cognitive tests, FDR correction for multiple comparisons was applied (denoted as pFDR).

Abbreviations: BF, basal forebrain; rho, Spearman's rank correlation coefficient; TMT‐B, Trail Making Test Part B; pFDR, false discovery rate–corrected *P*‐value; D‐KEFS CWIT, Delis–Kaplan Executive Function System Color‐Word Interference Test; RAVLT, Rey Auditory Verbal Learning Test; WMS‐III, Wechsler Memory Scale‐Third Edition; COWAT, Controlled Oral Word Association Test; MDS‐UPDRS III, Movement Disorder Society‐Unified Parkinson's Disease Rating Scale, Part III.

### Survival Analysis of iRBD Conversion to Synucleinopathy (PD or DLB) or Dementia‐Specific Synucleinopathy

**FIG. 1:**
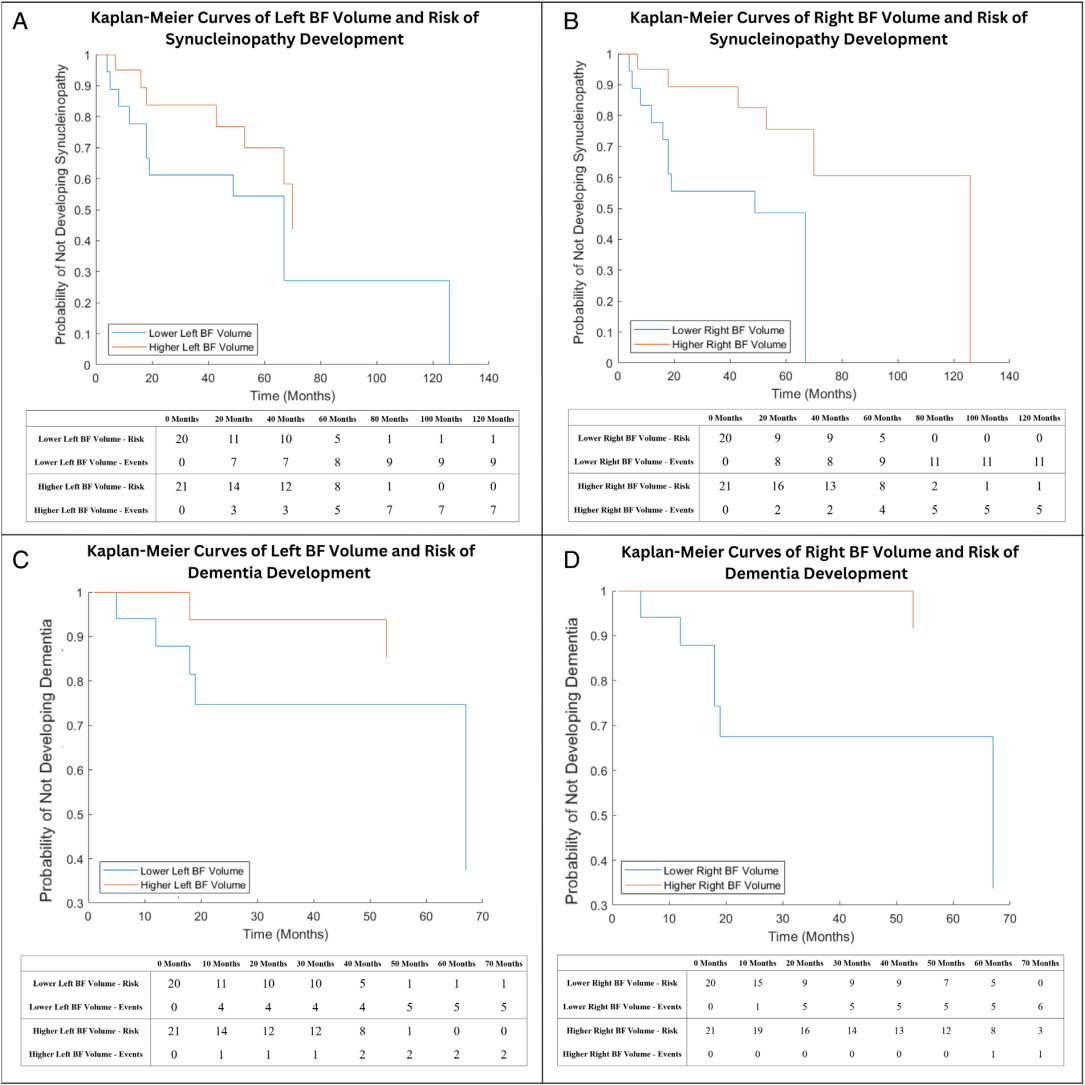
Kaplan–Meier survival curves illustrating the cumulative risk of phenoconversion based on basal forebrain (BF) volumes. Kaplan–Meier curves were constructed to visualize the cumulative risk of phenoconversion to α‐synucleinopathy (Parkinson's disease or dementia with Lewy bodies [DLB]) and dementia‐specific outcomes (DLB), with participants stratified into higher and lower BF volume groups using median volume across the iRBD (isolated rapid eye movement sleep behavior disorder) cohort as a cutoff (left BF: 305.92 mm^3^, right BF: 321.76 mm^3^). Risk tables below each plot show the number of participants who converted and those remaining at risk at each time point. (**A**) Left BF volume and the risk of conversion to α‐synucleinopathy. (**B**) Right BF volume and the risk of conversion to α‐synucleinopathy. (**C**) Left BF volume and the risk of dementia‐specific phenoconversion. (**D**) Right BF volume and the risk of dementia‐specific phenoconversion.

Cox proportional hazards models were used to examine the risk of phenoconversion to either α‐synucleinopathy (PD or DLB) or DLB specifically, based on baseline BF volume (Table 3). Kaplan–Meier curves that provide a visual representation of the cumulative risk over time are shown in Figure 1. Lower BF volumes were significantly associated with increased risk of disease conversion to PD or DLB. Specifically, each 1‐mm^3^ decrease in bilateral BF volume was associated with a 1.2% increased risk of α‐synucleinopathy conversion (HR = 1.012, 95% CI: 1.003–1.021, *P* = 0.008). For the left BF volume, the risk increased by 1.7% (HR = 1.017, 95% CI: 1.002–1.033, *P* = 0.025), whereas for the right BF volume, the increase was 2.0% (HR = 1.020, 95% CI: 1.005–1.035, *P* = 0.009). Regarding dementia‐specific phenoconversion, a 1‐mm^3^ decrease in bilateral BF volume was associated with an increased risk of 2.5% (HR = 1.025, 95% CI: 1.003–1.047, *P* = 0.025). For the left BF volume, the risk increased by 3.9% (HR = 1.039, 95% CI: 1.008–1.071, *P* = 0.014), whereas the right BF volume showed an increased risk of 2.8% (HR = 1.028, 95% CI: 1.001–1.055, *P* = 0.042).

**TABLE 3:** Cox proportional hazards models assessing the association between BF volume and risk of phenoconversion to α‐synucleinopathy or dementia in the iRBD cohort

Synucleinopathy development	Left BF volume	Right BF volume	Bilateral BF volume
Confidence interval (95%)	1.002, 1.033	1.005, 1.035	1.003, 1.021
Hazard ratio	1.017	1.020	1.012
*P*‐value	**0.025**	**0.009**	**0.008**
Log‐likelihood ratio tests	0.028	0.016	0.009

Dementia development	Left BF volume	Right BF volume	Bilateral BF volume
Confidence interval (95%)	1.008, 1.071	1.001, 1.055	1.003, 1.047
Hazard ratio	1.039	1.028	1.025
*P*‐value	**0.014**	**0.042**	**0.025**
Log‐likelihood ratio tests	0.001	0.010	0.001

*Notes*: Cox proportional hazards models were used to evaluate whether lower BF volume predicted increased risk of phenoconversion to α‐synucleinopathy (PD or DLB) and dementia (DLB) in the iRBD cohort. Results are presented separately for left, right, and bilateral BF volumes, including hazard ratios, 95% confidence intervals, *P*‐values, and log‐likelihood ratio test statistics. Values in bold indicate statistically significant predictive effects (*P < 0.05, two‐sided)*

Abbreviations: BF, basal forebrain; iRBD, isolated rapid eye movement sleep behavior disorder; DLB, dementia with Lewy bodies; PD, Parkinson's disease.

### Post hoc Survival Analysis Examining the Association between BF Volume and Disease‐Specific Phenoconversion in iRBD

To further investigate the potential lateralization effect observed in BF volume and its association with phenoconversion, we performed post hoc survival analyses focusing on the risk of phenoconversion to PD and DLB separately (Table 4). For these models, we compared individuals who converted to either PD or DLB with nonconverters, excluding those who developed the alternate diagnosis (PD converters vs. nonconverters excluding DLB converters; DLB converters vs. nonconverters excluding PD converters). In the PD‐specific analysis, right BF volume demonstrated a trend toward significance (HR = 1.017, 95% CI: 0.999–1.166, *P* = 0.065), with no significant association with left and bilateral BF volumes (HR = 1.009, *P* = 0.380; HR = 1.008, *P* = 0.120, respectively). In contrast, the DLB‐specific analysis revealed a significant association between BF volume and risk of phenoconversion. A 1‐mm^3^ decrease in the left BF volume was associated with a 4% increase in the risk of DLB conversion (HR = 1.040, 95% CI: 1.007–1.074, *P* = 0.017). Bilateral BF volume also demonstrated a significant relationship with DLB phenoconversion, where each 1‐mm^3^ decrease in bilateral BF volume was associated with a 2.9% increase in the risk of conversion to DLB (HR = 1.029, 95% CI: 1.004–1.054, *P* = 0.021). Right BF volume showed only a trend toward significance (HR = 1.057, 95% CI: 0.998–1.119, *P* = 0.058), suggesting a weaker, though still significant, association.

**TABLE 4:** Post hoc survival analysis examining the association between BF volume and specific phenoconversion to PD or DLB in iRBD

Parkinson's disease development	Left BF volume	Right BF volume	Bilateral BF volume
Confidence interval (95%)	0.989, 1.030	0.999, 1.166	0.998, 1.019
Hazard ratio	1.009	1.017	1.008
*P*‐value	0.380	0.065	0.120
Log‐likelihood ratio tests	0.641	0.198	0.307

DLB development	Left BF volume	Right BF volume	Bilateral BF volume
Confidence interval (95%)	1.007, 1.074	0.998, 1.119	1.004, 1.054
Hazard ratio	1.040	1.057	1.029
*P*‐value	**0.017**	0.0577	**0.021**
Log‐likelihood ratio tests	0.001	0.005	<0.001

*Notes*: Cox proportional hazards models were conducted post hoc to evaluate the relationship between BF volume and the risk of phenoconversion to PD or DLB, analyzed separately. In PD analysis, only participants who converted to PD were compared to nonconverters (DLB converters excluded). In DLB analysis, only participants who converted to DLB were compared to nonconverters (PD converters excluded). Hazard ratios, 95% confidence intervals, *P*‐values, and log‐likelihood ratio test statistics are reported for left, right, and bilateral BF volumes. Values in bold indicate statistically significant predictive effects (*P < 0.05, two‐sided)*.

Abbreviations: BF, basal forebrain; iRBD, isolated rapid eye movement sleep behavior disorder; DLB, dementia with Lewy bodies; PD, Parkinson's disease.

## Discussion

In this study, we examined the relationship between baseline BF volume and both cognitive deficits and the risk of progression to overt α‐synucleinopathy in individuals with iRBD. Despite the absence of group‐level differences between iRBD patients and HC, reduced BF volume within the iRBD cohort specifically was strongly associated with greater impairments in global cognition. Uniquely, baseline BF atrophy emerged as a significant predictor of phenoconversion, with smaller volumes conferring an elevated risk of progression, including dementia‐specific α‐synucleinopathies. Post hoc analyses examining disease‐specific outcomes indicated that the left and bilateral BF volumes were significantly associated with increased risk of conversion to DLB, whereas no BF regions were significantly associated with conversion to PD. Right BF volume exhibited only trend‐level associations with risk of conversion in both PD and DLB, suggesting a weaker or nonspecific relationship. These findings suggest a lateralized effect of BF degeneration, with left‐sided volume more closely linked to subsequent dementia onset in iRBD.

Although considerable evidence indicates that neurodegenerative changes within the BF occur early in the disease course of α‐synucleinopathies, our findings did not reveal significant differences in BF volumes in iRBD compared to controls. This may reflect the well established heterogeneity of iRBD, with variable pathological progression and a subset of nonprogressors, potentially contributing to attenuated group level differences. In line with this, previous studies involving iRBD cohorts with longer disease durations have reported significant BF volume loss in iRBD.^17^, ^18^ Multiple imaging modalities, including functional MRI and free‐water imaging, have also detected BF abnormalities in iRBD,^36^, ^37^ suggesting early microstructural or functional changes. Traditionally, pathological spread of α‐synuclein in iRBD is thought to follow a caudal–rostral trajectory,^38^, ^39^ initially affecting brainstem regions responsible for REM sleep regulation.^40^ However, more recent postmortem studies have also reported Lewy pathology within the BF in iRBD patients.^41^ This presence does not inherently imply structural atrophy, as neurodegeneration can manifest at a cellular level before volumetric loss becomes apparent.^41^ Therefore, the extent of BF atrophy may depend on the stage of pathological progression and overall impairment, influencing detectable group differences.^23^

Here, we add to the evidence that lower BF volumes are associated with cognitive impairment in iRBD.^42^ A similar relationship has been observed in PD, where BF volumes did not significantly differ from controls but were strongly associated with global cognitive performance.^43^ Cholinergic deficits and BF degeneration have been implicated in the pathophysiology of cognitive impairment across the spectrum of Lewy body diseases, which may be evident early in the disease stage.^12^, ^13^ Furthermore, BF atrophy has been directly linked to cortical cholinergic denervation and cognitive deficits in established PD.^15^ Although positron emission tomography (PET) imaging studies suggest that cholinergic dysfunction in iRBD is more subtle and variable^44^, ^45^, ^46^ compared to the drastic decline seen in DLB,^14^ a recent longitudinal study indicated a progressive reduction in cortical acetylcholinesterase activity in iRBD, which correlated with cognitive decline.^45^ Synthesizing these findings across disease groups and imaging modalities highlights a strong association between BF integrity and cognitive function, suggesting that disruptions in this pathway emerge in prodromal stages and may parallel progressive cortical cholinergic denervation.

Our results also point toward a lateralized pattern of BF degeneration that may be relevant for understanding disease‐specific trajectories in iRBD. Whereas reductions in right BF volume showed only trend‐level associations with phenoconversion to either PD or DLB, and were associated with more severe motor symptoms, left BF volume was significantly associated with increased risk of conversion to DLB specifically. Supporting this association between non‐motor symptoms and left BF atrophy, prior work in iRBD patients with MCI has shown selective reductions in left BF volume relative to HCs.^42^ Moreover, studies in DLB patients have demonstrated asymmetric cholinergic deficits, with greater reductions in left hemispheric vesicular acetylcholine transporter binding.^28^

Although relatively understudied, one possible explanation for the selective predictive value of left BF volume in DLB conversion may involve co‐pathological processes, particularly those associated with Alzheimer's disease (AD), where BF volumes in early‐diagnosed patients have also been predictive of cognitive decline.^47^, ^48^ Particularly, tau, amyloid, and α‐synuclein pathology have all been reported in the BF in both DLB and AD, with co‐occurring AD and Lewy body pathology associated with the most severe BF atrophy.^40^, ^49^ This raises the possibility that such co‐pathology could contribute to selective BF vulnerability in iRBD patients who later convert to DLB. While we did not observe significant differences in domain‐specific cognitive performance in our own iRBD cohort, prior studies in AD^50^ raise the possibility that lateralized BF degeneration may relate to domain‐specific cognitive decline, which could become more evident in larger or more cognitively impaired samples.

In summary, our findings add novel insight into identifying individuals at heightened risk of phenoconversion. Despite these promising findings, the relatively small cohort size and limited follow‐up period may constrain the generalizability of our survival analysis findings. Additionally, the absence of longitudinal imaging precludes direct assessment of progressive BF atrophy associated with disease conversion and limits our analysis to baseline BF volumes. Larger, multicenter studies with longitudinal imaging are needed to validate these associations and establish their broader clinical utility. Future work integrating the assessment of BF gray matter morphology with other predictive clinical^51^ and neuroimaging biomarker (free‐water imaging^36^ and [^18^F]FDG‐PET^52^) imaging could significantly refine patient stratification in iRBD. Furthermore, the incorporation of neurotransmitter‐specific imaging techniques could advance our understanding of early cholinergic dysfunction and, in turn, help develop composite imaging markers that could improve prognostic accuracy in clinical settings and facilitate the testing of disease‐modifying therapies.

## Author Roles

(1) Research project: A. Conception, B. Organization, C. Execution; (2) Statistical analysis: A. Design, B. Execution, C. Review and critique; (3) Manuscript: A. Writing of the first draft, B. Review and critique.

L.C.: 1A, 1B, 1C, 2A, 2B, 3A

A.K.: 1B, 1C, 2B, 2C, 3B

A.I.: 1B, 1C, 2B, 2C, 3B

J.A.: 1B, 1C, 2B, 2C, 3B

S.J.G.L.: 1B, 2C, 3B

E.M.: 1A, 1B, 2A, 2C, 3B

## Disclosures

**Ethical Compliance Statement:** Written informed consent was obtained from all patients, and ethical approval was obtained from the Sydney University Human Research Ethics Committee (HREC number 2013/HE000945). We confirm that we have read the journal's position on issues involved in ethical publication and affirm that this work is consistent with those guidelines.

**Funding Sources and Conflicts of Interest:** L.C. is the recipient of the Bierzonski Burczyk Foundation Postgraduate Research Scholarship. S.J.G.L. is supported by a National Health and Medical Research Council Leadership Fellowship (1195830) and has received research funding from the Michael J. Fox Foundation and the Australian Research Council, as well as consulting for Pharmaxis Ltd. E.M. is supported by a National Health and Medical Research Council Emerging Leadership Fellowship (2008565), the U.S. Department of Defense Congressionally Directed Medical Research Program Early Investigator Grant (PD220061) and the University of Sydney Horizon Fellowship. The authors declare no conflicts of interest relevant to this work.

**Financial Disclosures for the Previous 12 Months:** The authors declare that there are no additional disclosures to report.

## Supporting information


**Figure S1.** Basal forebrain (BF) segmentation using ScLimbic. T1‐weighted anatomical images were processed using the cross‐sectional stream of FreeSurfer (version 7.1.1). BF regions were segmented bilaterally using the ScLimbic deep‐learning toolbox. The segmented BF is highlighted in red for visualization.

**Data S1.** Overview of the residual correction method. Residual correction method was used to account for total intracranial volume in the gray matter volume analysis.

## Data Availability

The data that support the findings of this study are available from the corresponding author upon reasonable request.
