# Supplementary figures and images for: Basal Forebrain Volume Predicts Disease Conversion in Prodromal Synucleinopathy
Source: Mov Disord Clin Pract. 2025 Jul 22;13(1):198–207. doi: 10.1002/mdc3.70242 (PMC12839508; doi:10.1002/mdc3.70242)

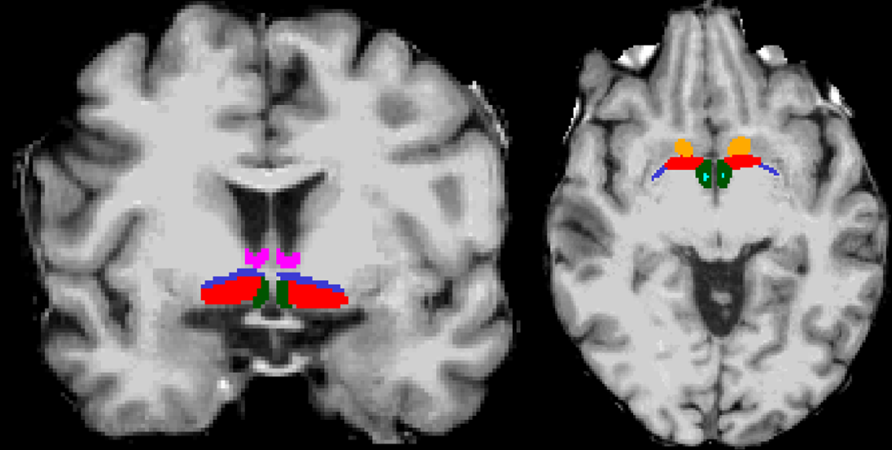

Supplement: Supplementary file 1 — Figure S1. Basal forebrain (BF) segmentation using ScLimbic. T1‐weighted anatomical images were processed using the cross‐sectional stream of FreeSurfer (version 7.1.1). BF regions were segmented bilaterally using the ScLimbic deep‐learning toolbox. The segmented BF is highlighted in red for visualization. [file MDC3-13-198-s002.png]
