# Supplementary material for: Basal Forebrain Volume Predicts Disease Conversion in Prodromal Synucleinopathy
Source: Mov Disord Clin Pract. 2025 Jul 22;13(1):198–207. doi: 10.1002/mdc3.70242 (PMC12839508; doi:10.1002/mdc3.70242)
Supplement: Supplementary file 2 — Data S1. Overview of the residual correction method. Residual correction method was used to account for total intracranial volume in the gray matter volume analysis. [file MDC3-13-198-s001.docx]

***Supplementary Methods***

*Residual Correction for Intracranial Volume (TIV):*

To account for inter-individual differences in total intracranial volume (TIV), we applied a residual correction method to the regional volumetric measures, following an approach originally described by Mathalon et al.^1^ This method is based on an analysis of covariance framework, aiming to eliminate the relationship between TIV and the volume of regions of interest (ROIs). Specifically, linear regression was performed with TIV as the independent variable and each ROI (e.g., left and right basal forebrain) as the dependent variable. The residuals from this regression capture the portion of the regional volume not explained by TIV. To preserve the original scale and maintain interpretability, we added the mean of the uncorrected regional volume back to the residuals. This results in TIV-adjusted volumes that retain biologically meaningful variation while minimizing the confounding effect of head size. The correction was implemented using custom MATLAB scripts, and the adjusted volumes were saved for use in all subsequent statistical analyses.

1. Mathalon DH, Sullivan EV, Rawles JM, Pfefferbaum A. Correction for head size in brain-imaging measurements. Psychiatry Res. 1993 Jun;50(2):121-39. doi: 10.1016/0925-4927(93)90016-b. Erratum in: Psychiatry Res 1994 Sep;55(3):179. PMID: 8378488.
